# Perinatal and Early-Life Exposures and Risk of Non-Hodgkin Lymphoma

**DOI:** 10.3390/cancers18162666

**Published:** 2026-08-18

**Authors:** George A. Cholack, Geffen Kleinstern, Dennis P. Robinson, Carrie A. Thompson, Timothy G. Call, Andrew L. Feldman, Melissa C. Larson, Raphael Mwangi, Stephen M. Ansell, Anne J. Novak, Neil E. Kay, Thomas M. Habermann, Wendy Cozen, Susan L. Slager, James R. Cerhan

**Affiliations:** 1 Department of Medicine, Division of Hematology, Mayo Clinic, Rochester, MN 55905, USA; 2 School of Public Health, Faculty of Social Welfare and Health Sciences, University of Haifa, Haifa 3498838, Israel; gkleinste@univ.haifa.ac.il; 3 Department of Quantitative Health Sciences, Mayo Clinic College of Medicine, 200 1st Street SW, Rochester, MN 55905, USA; 4 Department of Laboratory Medicine and Pathology, Mayo Clinic, Rochester, MN 55905, USA; 5 Division of Hematology/Oncology, Department of Medicine, University of California, Orange, CA 92868, USA

**Keywords:** early life, lymphomagenesis, etiology, perinatal, obesity

## Abstract

**Background:** Perinatal and early-life factors may influence non-Hodgkin lymphoma (NHL) risk, but study results have been mixed and exposure data for childhood ages are limited. Herein, we investigated associations of these exposures with risk of NHL and common subtypes. **Methods:** This case–control study included 2280 NHL cases and 2253 controls, enrolled from 2002 to 2014 at the Mayo Clinic Rochester. Self-reported perinatal and early-life exposures included maternal age at birth, birth order, birthweight, time breastfed, height and weight at ages 7, 12, and 18 years relative to peers, and age and weight when growth ceased. We used logistic regression to estimate odds ratios (ORs) and 95% confidence intervals (CIs), adjusting for design variables and potential confounders. Linear trend tests were performed for ordinal variables, and heterogeneity tests were performed to evaluate whether associations varied across four NHL subtypes. **Results:** After multivariable adjustment, greater birth weight (OR = 1.15 for quartile 4 vs. 1; *p*-trend = 0.04), weight at age 7 relative to peers (compared to average, OR = 1.05 for heavy and OR = 0.87 for thin; *p*-trend = 0.002), and weight when growth ceased (OR = 1.30 for quartile 4 vs. 1; *p*-trend < 0.001) were associated with increased NHL risk. An inverse association was observed for breastfeeding duration with NHL risk (OR = 0.77 for >6 months vs. never; 95% CI 0.61–0.97). Associations did not significantly vary by major NHL subtype (*p*-heterogeneity > 0.05). Other perinatal and early-life exposures were not associated with NHL risk. **Conclusions:** Greater body weight at birth and through childhood may be associated with increased NHL risk, potentially extending the life-course perspective on adiposity and lymphomagenesis, with implications for prevention.

## Simple Summary

Perinatal and early-life factors have been hypothesized to influence non-Hodgkin lymphoma (NHL) risk, but study results have been mixed and exposure data for childhood ages are limited. In this case–control study of 2280 NHL cases and 2253 controls, we assessed perinatal and early-life factors in association with risk of developing NHL later in life. Greater weight at birth, in childhood, and in late adolescence were all associated with greater risk of NHL. Breastfeeding duration of >6 months compared to never being breastfed was inversely associated with NHL risk. We did not observe any statistically significant associations for other perinatal and early life factors evaluated, and overall associations did not substantively vary by major NHL subtype. Excess body weight across the life course, including in early life, may influence risk of developing NHL later in life.

## 1. Introduction

Non-Hodgkin lymphoma (NHL) encompasses a broad group of lymphoid malignancies arising from either B- or T-lymphocytes or natural killer cells, and more than 100 subtypes have been described [1]. A total of 79,320 new cases of NHL are estimated to be diagnosed in 2025 [2]. The etiology of NHL remains incompletely understood, although some established risk factors include, but are not limited to, particular viruses (e.g., Epstein–Barr virus and hepatitis C), family history of hematologic malignancy, immunodeficiency (congenital or acquired), and autoimmune disease [3,4]. Identification of potentially modifiable risk factors remains a critical unmet need.

Perinatal and early-life exposures are associated with hormonal perturbations, growth trajectories, and varying microbial exposures, leading to the hypothesis that these factors may influence adult lymphomagenesis through various putative mechanisms, including proinflammatory cytokine milieu with concomitant immune dysregulation [5,6] and altered lymphatic system development based on early-life antigen exposures [7]. Associations of several perinatal and early-life factors with NHL risk have been explored in prior studies, and most can be summarized into one of these three exposure categories: maternal factors and/or breastfeeding status [8,9,10,11,12]; surrogates of infectious exposures in early-life (e.g., number of siblings, birth order, or other measures of crowding) [10,11,12,13,14,15,16,17]; and anthropometric measures (e.g., height and weight) across early lifespan [11,12,18,19,20,21,22,23,24]. Adult obesity has been recognized as a risk factor for NHL, which suggests that excess adiposity may contribute to lymphomagenesis plausibly through chronic inflammation; however, the literature for this association at earlier ages is limited [20,25]. Additionally, for many perinatal and early-life exposures, results are inconsistent across studies, likely in part due to variation in study design, outcome definitions, accuracy of measurements (e.g., self-report) and lymphoma subtypes included. This body of literature also has notable limitations, including evaluation in older cohorts predating contemporary lymphoma classification, limited assessment of subtype-specific associations for certain exposures, and varying degrees of adjustment for possible confounders. Most importantly, there is a paucity of the literature exploring the effect of anthropometric measurements in later childhood years (e.g., 7–12 years), which is a potentially important developmental window. To address these gaps, we conducted a case–control study to assess the hypothesized perinatal and early-life exposures with risk of NHL in adulthood, and evaluated heterogeneity by common NHL subtypes in exploratory analyses.

## 2. Methods

### 2.1. Study Population

This study was reviewed and approved by the Mayo Clinic Human Subjects Institutional Review Board; all participants provided written informed consent. Comprehensive details of this clinic-based case–control study conducted at the Mayo Clinic have been previously published [26]. Briefly, enrollment was prospectively offered to all consecutive cases of pathologically confirmed NHL or chronic lymphocytic leukemia (CLL)/small lymphocytic leukemia (SLL) who were within 9 months of initial diagnosis when presenting to the Mayo Clinic in Rochester, Minnesota. Additional eligibility criteria included the following: aged 18 years old and older; resident of Minnesota, Iowa, or Wisconsin at time of NHL diagnosis; no reported prior history of lymphoma, leukemia, or HIV/AIDS. All NHL cases were reviewed by a Mayo Clinic hematopathologist to verify the diagnosis and classified according to the World Health Organization classification of neoplastic diseases of the hematopoietic and lymphoid tissues [27]. Of the 4523 cases identified from 1 September 2002 to 31 August 2014, 3221 (71.2%) enrolled, 363 (8.0%) refused, 92 (2.0%) were never contacted after multiple attempts, and 847 (18.7%) failed to consent or complete the protocol after initial contact. We further excluded cases missing the risk factor questionnaire or with Hodgkin lymphoma, leaving 2280 eligible cases.

Controls were enrolled from Mayo Clinic Rochester patients who had prescheduled general medical examinations (e.g., examinations not intended for specific complaint or disease) in the general internal medicine department. Eligible controls included participants that had no history of lymphoma, leukemia, or HIV/AIDS. Additionally, controls needed to be at least 18 years of age or older, and a resident of Minnesota, Iowa, or Wisconsin at the time of the medical appointment. We used a computer program to randomly select controls, who were frequency-matched to cases based on the marginal distribution of 5-year age group, sex, and location of residence (distance from Rochester and urban/rural status). Of the 4363 identified controls from 1 September 2002 to 31 August 2014, 2489 (57.0%) participated, 1289 (29.5%) refused, and 585 (13.4%) failed to consent or complete the protocol after initial contact. We further excluded controls missing the risk factor questionnaire, leaving 2253 eligible controls.

### 2.2. Exposure Ascertainment

At the time of enrollment, participants completed a self-administered questionnaire that ascertained information regarding demographic characteristics (age, sex, residence, educational status); family history; smoking history (never, former, or current use); alcohol history (never, former, or current use); history of autoimmune disease (B- and T-cell-mediated autoimmune diseases); history of allergies; body mass index (BMI) in young adulthood; and recreational sun exposure (hours per week). These data were harmonized with exposure definitions developed for the International Lymphoma Epidemiology (Interlymph) Consortium NHL Subtypes Project [3].

For perinatal exposures, participants were asked to provide information about their own gestation and birth, including their mother’s age when they were born; their birth order (first child, second child, third child, or 4+); birth weight (in kilograms); birth type (singleton or twin); pre-term birth (defined as 4 or more weeks premature); if their mother had eclampsia or preeclampsia during pregnancy; personal history of neonatal jaundice; and whether they were breast fed and for how long (never, 1–6 months, and >6 months). For early-life exposures, participants were asked to compare their height at age 7 years (about 1st grade), 12 years (about 6th grade) and 18 years (about 12th grade) relative to other girls/boys their age (short, average, or tall) and their relative weight (thin, average, or heavy) at those ages; the age when they stopped getting taller (<17, 17–18, and 19+); and how much they weighed at that time (in kilograms). BMI at age 18 was calculated based on the height and weight that participants reported they were at age 18.

### 2.3. Statistical Analysis

We used separate binary logistic regressions to estimate odds ratios (ORs) and 95% confidence intervals (CIs) for associations of perinatal and early-life exposures with risk of developing NHL overall and with each selected NHL subtype individually: diffuse large B-cell lymphoma (DLBCL), follicular lymphoma (FL), CLL/SLL, and all remaining subtypes combined as “Other NHL” due to limited sample sizes of these other subtypes. All estimates were adjusted for age upon enrollment, sex, and proximity to Rochester, Minnesota (<120 miles versus ≥120 miles). We additionally adjusted for education (categories: up to high school graduation, education beyond high school/some college, college graduate/1+ years of graduate or professional school, missing), any allergy (harmonized from plant, food, animal, dust, insect, and/or mold), reported family history of NHL in first-degree relative, reported history of B-cell-mediated autoimmune disease (rheumatoid arthritis, Sjögren’s syndrome, or systemic lupus erythematosus), reported history of T-cell-mediated autoimmune disease (Celiac’s disease, myositis, inflammatory bowel disease, Crohn’s disease, ulcerative colitis, or type 1 diabetes mellitus), and recreational sun exposure as all of these factors have been shown to be associated with incident NHL risk [3]. Additionally, all analyses except birth weight and age and weight when they stopped growing taller were further adjusted for reported BMI at 18 years of age. These three exposures were also categorized into sex-specific quartiles. Participants with missing data for a given exposure were excluded for that given logistic regression model. For ordinal variables, we also assessed for a linear trend across levels of a given variable. Finally, to assess heterogeneity among the four lymphoma subtype categories, we performed a Wald test for heterogeneity with degrees of freedom based on the number of categories for a given variable. To assess whether the BMI at age 18 may represent a downstream variable on the causal pathway between childhood body size and NHL risk, we conducted a post hoc sensitivity analysis in which all models were re-estimated without adjustment for BMI at age 18. All reported *p*-values are two-sided, and differences were considered statistically significant at *p* < 0.05. All analyses were performed using SAS version 9.4.

## 3. Results

### 3.1. Baseline Characteristics

Characteristics of cases and controls are summarized in Table 1. Missingness by case–control status is summarized in Supplemental Table S1. Briefly, the median age at diagnosis for NHL cases (n = 2280) was 63 years (range: 18–91); 41.5% were female, and 98.6% identified as Non-Hispanic White. For controls (n = 2253), the median age upon enrollment was 64 years (range: 18–93); 47.2% were female, and 97.9% identified as Non-Hispanic White. The majority of cases (68.3%) and controls (79.3%) were from Minnesota. Controls also had modestly higher educational level (45% of controls had graduated college compared to only 34.6% of cases). The most common lymphoma subtypes were CLL/SLL (28.2%), FL (23.2%), and DLBCL (18.2%).

### 3.2. Perinatal Exposures and Adult NHL Risk

Cases and controls had similar proportions of missing data across perinatal exposures, with <30% missing for most variables, and were highest for breast feeding duration (~47%). Missingness was not associated with case–control status or with covariates of interest, apart from breastfeeding duration. Missingness of breastfeeding duration was not associated with case–control status, although it was associated with several measured participant characteristics (Supplemental Tables S2 and S3). Associations of perinatal factors with NHL risk in adulthood (overall and by NHL subtype) are summarized in Table 2. After multivariable adjustment, we did not observe statistically significant associations or trends with NHL risk for the following variables: maternal age, birth order, birth type (singleton vs. twin), pre-term birth, eclampsia/preeclampsia, and history of neonatal jaundice. There was a statistically significant positive, monotonic trend across increasing quartiles of birth weight for NHL overall (*p*-trend = 0.04) and DLBCL (*p*-trend = 0.04), but not for other NHL, FL, or CLL/SLL. Compared to never being breastfed, breastfeeding for >6 months was associated with a reduced risk of NHL overall (OR 0.77, 95% CI 0.61–0.97) and for CLL/SLL (OR 0.63, 95% CI 0.43–0.91). The ORs for breastfeeding 1–6 months were <1, although they were not statistically significant nor was the trend test. No significant associations were observed for breastfeeding status with risk of DLBCL, FL, or other NHL. Heterogeneity tests by lymphoma subtypes were not significant for any perinatal exposure (all *p*-heterogeneity > 0.05). In post hoc sensitivity analysis, point estimates and trends remained consistent with those of the primary analysis when BMI at age 18 was not included as a covariate.

### 3.3. Early-Life Exposures and Adult NHL Risk

Cases and controls had similar proportions of missing data across early-life exposures, with <25% missing for most variables. Missingness was highest for reported weight when growth ceased (roughly 29%), but was not associated with case–control status or with covariates of interest. Associations of early-life factors with NHL risk in adulthood (overall and by NHL subtype) are summarized in Table 3. We did not observe significant associations or trends for relative height at ages 7 and 12, relative weight at age 12, or relative weight at age 18. We did not observe significant associations or trends for reported age at which growth ceased, except for CLL/SLL (*p*-trend = 0.04). In contrast, significant positive trends were observed for increasing reported weight quartiles when growth ceased with risk of overall NHL (*p*-trend < 0.001), DLBCL (*p*-trend = 0.004), CLL/SLL (*p*-trend = 0.01), and other NHL (*p*-trend = 0.03). A statistically significant, monotonic trend was also observed for increasing relative weight at age 7 in relation to overall NHL risk (*p*-trend = 0.002), although individual weight category estimates were not statistically significant. For “other NHL,” significant trends were observed for relative weight at age 7 and weight when growth ceased (both *p*-trend < 0.001). A significant trend was observed between relative height at age 18 and risk of FL (*p*-trend = 0.02), but no consistent associations were observed for the other subtypes. Tests for heterogeneity by lymphoma subtypes were not statistically significant for any early-life exposure (all *p*-heterogeneity > 0.05). In post hoc sensitivity analysis, point estimates and trends remained consistent with those of the primary analysis when BMI at age 18 was not included as a covariate.

## 4. Discussion

In this large clinic-based case–control study, we observed several key findings. First, reported higher weight when growth ceased was associated with increased non-Hodgkin lymphoma (NHL) risk overall and for most NHL subtypes. Second, greater birth weight and relative weight at age seven were associated with increased overall NHL risk with trends across sex-specific quartiles and categories, respectively. Third, we observed a suggestive inverse association of longer breastfeeding duration with overall adulthood NHL risk, although the trend test was not statistically significant. Finally, we did not observe meaningful associations between other investigated perinatal and early-life exposures and risk of NHL in adulthood, overall or by NHL subtype.

Over the past two decades, most of the literature supports a modest positive association between adulthood BMI and incident NHL risk. In a 2019 meta-analysis, the World Cancer Research Fund Network and American Institute for Cancer Research demonstrated that adulthood BMI and BMI in early adulthood (18–21 years) were associated with a relative risk of 1.05 (1.03–1.08) and 1.12 (95% CI 1.09–1.37) per 5 kg/m^2^ in BMI, respectively [20]. A 2018 meta-analysis of 22 prospective cohorts reported similar results [25]. In contrast, the Interlymph Consortium conducted a large, pooled case–control study and reported a pooled OR 1.00 (95% CI 0.70–1.41) for NHL overall, but they did observe an OR 1.80 (95% CI 1.24–2.62) for diffuse large B-cell lymphoma (DLBCL) [21]. Other case–control and cohort studies have also shown similar results for associations between increasing late-adolescent and/or adult BMI with risk for all NHL [19,22,23], DLBCL [19,22,23], follicular lymphoma (FL) [19,22], and chronic lymphocytic leukemia and small lymphocytic leukemia (CLL/SLL) [19,22,23]. Although our FL-specific results slightly differed from other studies [19,22], our remaining associations of increasing reported weight (once growth ceased) with increasing risk of NHL overall, DLBCL, and CLL are concordant with other studies [19,20,21,22,23,25,28]. Consistent with other studies [18,28], our findings support the notion that obesity (potential surrogate for adiposity) earlier in life (e.g., young adulthood) may confer increased NHL risk and represent a potential opportunity for earlier primary NHL prevention.

Our finding that higher birth weight was associated with increased overall NHL risk is concordant with prior studies [10,12,24]. In contrast, there is a paucity of the literature reporting associations of body size in later childhood (7–12 years) and subsequent NHL risk in adulthood. While several cohort studies have reported positive associations between weight/body size at age 10 years and NHL risk [11,18,24], only one of these studies [18] additionally reported a linear association between heavier childhood somatotypes (perceived body shape) at age 5 and increased NHL risk in adulthood. In our study, we observed a significant trend association of greater relative weight at 7 with increased NHL risk, although most subtype-specific associations were not statistically significant and require cautious interpretation. This finding will require replication, as this result may indicate a non-linear relationship (given the non-significant categorical odds ratios comparing “heavy” and “thin” groups to “average”), lower power in the non-average categories, or a genuine linear association. If the latter, this association suggests that adiposity earlier in life might contribute to increased NHL risk in adulthood, warranting further study. The relationship between obesity, a recognized proinflammatory state, and NHL risk is complex, but several mechanisms underlying such an association have been proposed, including altered levels of B-lymphocyte stimulatory cytokines, altered adipokines, increased IGF-1 production inhibiting B-lymphocyte apoptosis, insulin resistance favoring cell proliferation, and genetic polymorphisms in obesity-related genes, such as leptin [29]. Given the increasing prevalence of pediatric obesity in the United States [30], further research into early-life obesity/adiposity as a potentially modifiable risk factor for non-Hodgkin lymphomagenesis is warranted.

There are limited data evaluating breastfeeding in relation to NHL risk in adulthood. Most case–control studies have focused on the association of breastfeeding with risk of developing childhood hematologic malignancy, particularly lymphoma and leukemia [31,32,33,34,35]. The majority of these studies demonstrated a protective association with breastfeeding [32,33,34]; however, Hardell et al. [35] and Bener et al. [31] both observed an increased risk of developing pediatric NHL. Only two prospective cohort studies have explored the association of breastfeeding with NHL risk in adulthood, but these studies [8,9] only included female participants. Neither Costas et al. [8] nor Tanaka et al. [9] observed statistically significant associations of breastfeeding with NHL risk in adulthood, but there was a suggestive inverse trend noted in the latter study with an observed HR 0.80 (95% CI 0.41–1.57) for participants who had been breastfed relative to those who were never breastfed. Our results are broadly concordant with these studies [8,9], as we observed a lower risk of NHL in adults with breastfeeding longer than 6 months, as well as a lower, albeit not statistically significant lower, risk for short (1–6 months) duration. As the trend test was also not statistically significant, we cannot clearly distinguish a dose–response association versus a non-linear threshold effect above 6 months, which will require larger studies to determine. Based on other work, potential biologically plausible mechanisms underlying a protective association include promotion of immune tolerance in early-life, transfer of secretory IgA through breast milk, and promotion of optimal microbiome development through human milk oligosaccharides [36,37,38,39]. To our knowledge, this is the first study to explore this association in a study that include data on males and sufficient sample size to estimate associations for the most common NHL subtypes [8,9]. Our results would benefit from replication in larger studies to achieve greater statistical power, particularly for rarer NHL subtypes.

Finally, the lack of significant associations for other perinatal and early-life factors, particularly birth order, warrants consideration. Numerous studies have examined proxies of early-life exposure to infectious agents to assess the hygiene hypothesis, which proposes that reduced microbial exposure in early-life may impair adaptive immune system development and thus increase subsequent risk of lymphoproliferative disease [7]. However, adolescent/young adult Hodgkin lymphoma appears to be an exception to this pattern, as greater oral microbial exposure has been suggested to confer a protective effect, potentially through modulation of Th2/Th1 cytokine balance [40]. Lower birth order has been proposed as a surrogate for reduced early-life microbial exposure. Overall, the literature exploring associations of birth order with risk of subsequent NHL at any point in life has been mixed, with some studies reporting increased risk with lower birth order [10,14,41,42,43], higher birth order [13,44,45], or no association [15,46]. Our study’s result aligns with two of these studies [15,46], which suggests that birth order may be a suboptimal surrogate for early-life microbial exposure. It is also worth noting that the discordant findings amongst these studies are likely in part due to variances in study population, lymphoma subtypes analyzed, and potential biases such as recall bias.

This study has several notable strengths, including large sample size; expert pathology review and classification according to the World Health Organization criteria [27]; the first study to explore associations with self-reported early-life factors at age 7 and 12; and assessment of confounding by adjusting for several factors known to influence lymphomagenesis. This study is not without limitations. First, the case–control study design has the potential for recall and selection biases. Self-reporting of the exposures can lead to either differential or nondifferential misclassification, thus impacting study validity. Differential misclassification by case–control status could bias estimates of associations in either direction, while nondifferential misclassification (e.g., random recall error) would generally be expected to attenuate the observed associations. Reliability of self-reported exposures early in life is also a potential concern as is validity, and this will likely vary by self-reported exposure. However, some studies have shown good concordance between participant recall of such exposures and recorded measurements, including birth weight and height and weight in early life [47,48]. Additionally, self-reported, relative height and weight categories may not accurately reflect adiposity. There was substantial missingness for some exposures (e.g., breastfeeding status and duration) and this would lower power if missing at random and could introduce bias in either direction if not missing at random. For breastfeeding status, participants with missing data differed from those with available data with respect to several characteristics, including age and sex. These differences suggest that missingness was not completely at random for this exposure, raising the possibility of selection bias; therefore, findings for breastfeeding require cautious interpretation. Residual confounding by known and unknown factors is another important consideration. While we were able to adjust for a variety of potential confounders such as adult education level, several other known factors related to maternal or childhood environments were not measured. Other limitations include inability to assess rarer lymphoma subtypes, challenging interpretation of the “Other NHL” group due to disease heterogeneity, inability to assess relationship with number of siblings (total, older, and younger), and potentially limited external validity to non-White populations and populations in different geographic regions. Finally, due to the multiple comparisons analyzed in this study, these results should be considered as hypothesis-generating, and statistically significant associations require cautious interpretation and replication, ideally in a prospective setting.

## 5. Conclusions

While requiring further validation, greater body weight at birth and through childhood may influence NHL risk, extending the life-course perspective on adiposity and lymphomagenesis. As obesity is a modifiable risk factor and its prevalence continues to increase, particularly in younger populations, prevention may need to extend to begin earlier in life.

## Figures and Tables

**Table 1:** Characteristics of cases and controls in the Mayo Clinic case–control study of NHL (n (%)).

	Controls	Cases
	N = 2253	N = 2280
**Age (years)**		
Mean (standard deviation)	61.6 (13.2)	61.7 (13.1)
Median	64	63
Q1, Q3 (Quartile)	54.0, 71.0	54.0, 71.0
Range	18.0–93.0	18.0–91.0
**Sex**		
Female	1063 (47.2)	946 (41.5)
Male	1190 (52.8)	1334 (58.5)
**Reported Race/Ethnicity**		
Non-Hispanic White	2206 (97.9%)	2247 (98.6%)
Non-Hispanic Black	8 (0.4%)	4 (0.2%)
Non-Hispanic Asian	7 (0.3%)	10 (0.4%)
Non-Hispanic Other	10 (0.4%)	1 (0.0%)
Hispanic (all races)	22 (1.0%)	18 (0.8%)
**Residence**		
Iowa	268 (11.9)	405 (17.8)
Minnesota	1787 (79.3)	1558 (68.3)
Wisconsin	196 (8.7)	298 (13.1)
Missing	2	19
**Education**		
Some high school	517 (22.9)	551 (24.2)
Beyond high school	608 (27.0)	607 (26.6)
College Graduate	1020 (45.3)	788 (34.6)
Missing/other	108	334
**Mayo Registration**		
≤1991	1366 (60.8)	748 (33.8)
>1991	881 (39.2)	1464 (66.2)
**Family history NHL**		
No	2162 (96.2)	2120 (93.2)
Yes	86 (3.8)	154 (6.8)
Missing	5	6
**Smoking history**		
Never	1267 (57.7)	1202 (54.2)
Former	789 (36.0)	819 (36.9)
Current	138 (6.3)	196 (8.8)
Missing	59	63
**Alcohol history**		
Never	446 (20.1)	528 (23.4)
Former	454 (20.4)	504 (22.4)
Current	1321 (59.5)	1221 (54.2)
Missing	32	27
**History B-cell autoimmune disease**		
No	2114 (94.7)	2083 (92.5)
Yes	118 (5.3)	168 (7.5)
Missing	21	29
**History T-cell autoimmune disease**		
No	2134 (95.5)	2155 (95.6)
Yes	101 (4.5)	99 (4.4)
Missing	18	26
**History of any allergy**		
No	1515 (67.2)	1670 (73.3)
Yes	738 (32.8)	608 (26.7)
Missing	0	2
**BMI at age 18 years**		
≤18.5	234 (10.6)	181 (8.1)
18.5–22.5	1157 (52.2)	1130 (50.7)
22.5–25	520 (23.5)	512 (23.0)
25–30	245 (11.1)	346 (15.5)
≥30	59 (2.7)	59 (2.6)
Missing	38	52
**Sun Exposure (Quartile)**		
Q1/Q2	1075 (48.9)	921 (47.0)
Q3/Q4	1124 (51.1)	1037 (53.0)
Missing	54	322
**NHL Subtype**		
Control	2253 (100.0)	0 (0.0)
CLL/SLL	0	644 (28.2)
DLBCL	0	414 (18.2)
FL	0	528 (23.2)
MCL	0	127 (5.6)
MZL	0	170 (7.5)
TCL	0	119 (5.2)
Other	0	278 (12.2)

Abbreviations: BMI, body mass index; CLL, chronic lymphocytic leukemia; DLBCL, diffuse large B-cell lymphoma; FL, follicular lymphoma; MCL, mantle cell lymphoma; MZL, marginal zone lymphoma; NHL, non-Hodgkin lymphoma; SLL, small lymphocytic leukemia; TCL, T-cell lymphoma.

**Table 2:** Associations between self-reported perinatal exposures and risk of NHL and NHL subtypes (ORs and 95% CIs) *.

			All NHL		DLBCL		FL		CLL/SLL		Other NHL	
Exposure ^†^	Category	Controls	Cases	OR (95% CI)	Cases	OR (95% CI)	Cases	OR (95% CI)	Cases	OR (95% CI)	Cases	OR (95% CI)
Maternal age (years)	Q1 (≤23)	427	429	Reference	78	Reference	111	Reference	112	Reference	128	Reference
	Q2 (23–<27)	511	526	1.09 (0.89–1.34)	93	1.14 (0.78–1.67)	122	0.91 (0.66–1.26)	157	1.34 (0.98–1.84)	154	1.03 (0.77–1.40)
	Q3 (27–<32)	537	531	1.05 (0.86–1.29)	98	1.15 (0.79–1.68)	133	0.94 (0.68–1.30)	145	1.18 (0.86–1.61)	155	0.99 (0.74–1.34)
	Q4 (≥32)	588	585	1.03 (0.84–1.25)	108	1.13 (0.78–1.64)	130	0.85 (0.61–1.17)	162	1.15 (0.84–1.56)	185	1.00 (0.74–1.34)
				*p*-trend = 0.39		*p*-trend = 0.48		*p*-trend = 0.16		*p*-trend = 0.47		*p*-trend = 0.38
Birth order	1st	752	737	Reference	140	Reference	169	Reference	197	Reference	231	Reference
	2nd	605	617	0.99 (0.84–1.18)	111	0.97 (0.71–1.32)	134	0.95 (0.71–1.26)	190	1.10 (0.85–1.42)	182	0.94 (0.73–1.20)
	3rd	381	366	0.94 (0.77–1.14)	59	0.81 (0.56–1.19)	91	0.94 (0.67–1.30)	104	1.07 (0.79–1.44)	112	0.93 (0.70–1.25)
	4+	478	535	1.08 (0.91–1.30)	98	1.00 (0.72–1.39)	128	1.12 (0.84–1.51)	148	1.21 (0.92–1.59)	161	0.89 (0.68–1.16)
				*p*-trend = 0.09		*p*-trend = 0.39		*p*-trend = 0.08		*p*-trend = 0.15		*p*-trend = 0.26
Birth weight (kilograms) ^‡^	Q1	267	255	Reference	43	Reference	64	Reference	74	Reference	74	Reference
	Q2	348	317	0.90 (0.70–1.16)	47	0.95 (0.57–1.59)	71	0.96 (0.62–1.47)	98	0.84 (0.58–1.23)	101	0.90 (0.62–1.33)
	Q3	298	298	1.04 (0.81–1.35)	46	1.14 (0.68–1.89)	80	1.25 (0.82–1.89)	91	0.96 (0.65–1.40)	81	0.96 (0.65–1.41)
	Q4	343	385	1.15 (0.90–1.47)	74	1.46 (0.91–2.35)	89	1.17 (0.78–1.77)	98	0.88 (0.60–1.28)	124	1.23 (0.85–1.78)
				*p*-trend = 0.04		*p*-trend = 0.04		*p*-trend = 0.17		*p*-trend = 0.39		*p*-trend = 0.06
Birth type	Singleton	2189	2227	Reference	407	Reference	517	Reference	626	Reference	677	Reference
	Twin+	46	40	0.80 (0.50–1.30)	5	0.71 (0.28–1.85)	9	0.60 (0.25–1.48)	13	1.02 (0.53–1.98)	13	0.67 (0.31–1.45)
Gestational birth	No	2156	2196	Reference	399	Reference	511	Reference	619	Reference	667	Reference
	Yes	64	65	1.13 (0.77–1.67)	11	0.94 (0.44–2.04)	14	1.14 (0.61–2.15)	20	1.39 (0.79–2.42)	20	1.03 (0.57–1.85)
Eclampsia/ Preeclampsia	No	1546	1490	Reference	258	Reference	363	Reference	409	Reference	460	Reference
	Yes	28	25	1.00 (0.52–1.91)	5	1.35 (0.44–4.14)	7	0.75 (0.25–2.27)	7	1.05 (0.38–2.90)	6	0.87 (0.31–2.42)
Neonatal jaundice	No	1417	1340	Reference	228	Reference	327	Reference	373	Reference	412	Reference
	Yes	63	64	1.08 (0.71–1.64)	14	1.61 (0.78–3.35)	19	1.55 (0.84–2.85)	11	0.75 (0.34–1.63)	20	1.01 (0.53–1.92)
Time breastfed	Never	583	598	Reference	117	Reference	136	Reference	168	Reference	177	Reference
	1–6 months	342	319	0.90 (0.72–1.11)	56	0.76 (0.50–1.14)	79	1.08 (0.76–1.52)	84	0.80 (0.58–1.10)	100	0.93 (0.68–1.28)
	>6 months	285	258	0.77 (0.61–0.97)	48	0.84 (0.54–1.29)	72	1.01 (0.69–1.48)	65	0.63 (0.43–0.91)	73	0.79 (0.55–1.13)
				*p*-trend = 0.14		*p*-trend = 0.11		*p*-trend = 0.50		*p*-trend = 0.11		*p*-trend = 0.34

Abbreviations: BMI, body mass index; CLL, chronic lymphocytic leukemia; DLBCL, diffuse large B-cell lymphoma; FL, follicular lymphoma; NHL, non-Hodgkin lymphoma; SLL, small lymphocytic leukemia. * Adjusted for age, sex, proximity to Rochester, Minnesota (<120 miles versus ≥120 miles), education level, any allergy, reported family history of NHL in first-degree relative, self-reported history of B- and T-cell-mediated autoimmune disease, smoking history, alcohol history, and recreational sun exposure. All analyses except birth weight were further adjusted for reported BMI at 18 years of age. ^†^ Self-reported. ^‡^ Birth weight was categorized into sex-specific quartiles: men, <3.18 kg, 3.18–3.62 kg, 3.63–4.03 kg, and >4.03 kg; women, <2.92 kg, 2.92–3.31 kg, 3.32–3.63 kg, and >3.63 kg.

**Table 3:** Associations between self-reported early life exposures and risk of NHL and NHL subtypes (ORs and 95% CIs) *.

			All NHL		DLBCL		FL		CLL/SLL		Other NHL	
Exposure ^†^	Category	Controls	Cases	OR (95% CI)	Cases	OR (95% CI)	Cases	OR (95% CI)	Cases	OR (95% CI)	Cases	OR (95% CI)
Relative Height at 7 years	Short	361	359	0.97 (0.81–1.17)	59	0.99 (0.70–1.39)	91	0.97 (0.72–1.31)	90	0.91 (0.69–1.21)	119	1.05 (0.80–1.36)
	Average	1425	1470	Reference	265	Reference	333	Reference	421	Reference	451	Reference
	Tall	381	362	0.92 (0.77–1.10)	69	1.07 (0.77–1.50)	79	0.86 (0.64–1.17)	108	1.00 (0.76–1.30)	106	0.87 (0.66–1.14)
				*p*-trend = 0.15		*p*-trend = 0.34		*p*-trend = 0.27		*p*-trend = 0.25		*p*-trend = 0.20
Relative Height at 12 years	Short	367	363	0.99 (0.83–1.19)	61	1.07 (0.76–1.51)	84	0.86 (0.63–1.16)	93	0.99 (0.75–1.31)	125	1.12 (0.86–1.46)
	Average	1311	1326	Reference	234	Reference	324	Reference	366	Reference	402	Reference
	Tall	530	522	0.99 (0.84–1.16)	99	1.16 (0.86–1.56)	102	0.73 (0.56–0.97)	170	1.23 (0.97–1.55)	151	0.94 (0.73–1.19)
				*p*-trend = 0.35		*p*-trend = 0.41		*p*-trend = 0.02		*p*-trend = 0.14		*p*-trend = 0.33
Relative Height at 18 years	Short	366	296	0.83 (0.68–1.00)	54	0.89 (0.62–1.28)	69	0.76 (0.55–1.04)	79	0.87 (0.65–1.17)	94	0.81 (0.61–1.09)
	Average	1372	1425	Reference	249	Reference	344	Reference	395	Reference	437	Reference
	Tall	486	504	0.98 (0.83–1.15)	93	1.15 (0.85–1.55)	103	0.79 (0.60–1.05)	159	1.18 (0.93–1.49)	149	0.90 (0.70–1.14)
				*p*-trend = 0.27		*p*-trend = 0.46		*p*-trend = 0.04		*p*-trend = 0.25		*p*-trend = 0.24
Relative Weight at 7 years	Thin	743	652	0.87 (0.75–1.02)	119	0.96 (0.73–1.28)	162	0.93 (0.72–1.19)	189	0.97 (0.77–1.22)	182	0.72 (0.58–0.91)
	Average	1313	1401	Reference	250	Reference	317	Reference	384	Reference	450	Reference
	Heavy	144	157	1.05 (0.80–1.38)	28	0.95 (0.58–1.57)	35	0.91 (0.57–1.45)	48	1.28 (0.86–1.89)	46	0.97 (0.65–1.46)
				*p*-trend = 0.002		*p*-trend = 0.08		*p*-trend = 0.18		*p*-trend = 0.10		*p*-trend < 0.001
Relative Weight at 12 years	Thin	678	625	0.96 (0.82–1.12)	102	0.91 (0.67–1.23)	157	1.06 (0.82–1.38)	185	1.09 (0.86–1.38)	181	0.85 (0.68–1.08)
	Average	1267	1306	Reference	241	Reference	287	Reference	362	Reference	416	Reference
	Heavy	269	296	1.06 (0.85–1.31)	56	1.04 (0.70–1.53)	73	1.02 (0.72–1.46)	83	1.12 (0.82–1.54)	84	0.99 (0.72–1.37)
				*p*-trend = 0.07		*p*-trend = 0.05		*p*-trend = 0.37		*p*-trend = 0.36		*p*-trend = 0.02
Relative Weight at 18 years	Thin	583	547	1.00 (0.84–1.18)	87	1.04 (0.75–1.43)	133	0.96 (0.73–1.28)	161	1.17 (0.91–1.51)	166	0.89 (0.70–1.15)
	Average	1445	1481	Reference	274	Reference	330	Reference	420	Reference	457	Reference
	Heavy	194	205	0.88 (0.67–1.15)	38	0.85 (0.53–1.38)	56	1.10 (0.71–1.70)	50	0.80 (0.52–1.22)	61	0.77 (0.51–1.16)
				*p*-trend = 0.12		*p*-trend = 0.04		*p*-trend = 0.42		*p*-trend = 0.28		*p*-trend = 0.16
Age when height stopped growing (years) ^‡^	Q1	366	350	Reference	59	Reference	79	Reference	122	Reference	90	Reference
	Q2	353	374	0.98 (0.78–1.23)	75	1.11 (0.73–1.68)	83	0.96 (0.65–1.40)	97	0.83 (0.59–1.16)	119	1.21 (0.85–1.72)
	Q3	525	536	1.02 (0.83–1.26)	95	1.00 (0.68–1.48)	138	1.16 (0.83–1.63)	141	0.82 (0.60–1.11)	162	1.18 (0.86–1.64)
	Q4	474	442	0.89 (0.72–1.10)	81	0.90 (0.60–1.35)	99	0.83 (0.58–1.20)	122	0.75 (0.55–1.03)	140	1.11 (0.80–1.55)
				*p*-trend = 0.34		*p*-trend = 0.47		*p*-trend = 0.50		*p*-trend = 0.04		*p*-trend = 0.21
Weight when height stopped growing (quartiles by kilograms) ^§^	Q1	386	333	Reference	57	Reference	87	Reference	82	Reference	107	Reference
	Q2	395	328	1.00 (0.80–1.26)	50	0.75 (0.47–1.19)	74	0.97 (0.67–1.41)	107	1.27 (0.90–1.79)	97	0.91 (0.64–1.28)
	Q3	378	439	1.38 (1.11–1.72)	84	1.50 (1.00–2.25)	101	1.27 (0.89–1.82)	130	1.54 (1.10–2.16)	124	1.25 (0.90–1.73)
	Q4	424	459	1.30 (1.04–1.62)	88	1.41 (0.95–2.10)	105	1.08 (0.76–1.56)	124	1.33 (0.94–1.87)	142	1.27 (0.92–1.74)
				*p*-trend < 0.001		*p*-trend = 0.004		*p*-trend = 0.11		*p*-trend = 0.01		*p*-trend = 0.03

Abbreviations: BMI, body mass index; CLL, chronic lymphocytic leukemia; DLBCL, diffuse large B-cell lymphoma; FL, follicular lymphoma; NHL, non-Hodgkin lymphoma; SLL, small lymphocytic leukemia. * Adjusted for age, sex, proximity to Rochester, Minnesota (<120 miles versus ≥120 miles), education level, any allergy, reported family history of NHL in first-degree relative, self-reported history of B- and T-cell-mediated autoimmune disease, smoking history, alcohol history, and recreational sun exposure. All analyses except age and weight when height stopped growing were further adjusted for reported BMI at 18 years of age. ^†^ Self-reported. ^‡^ Age when height stopped growing was categorized into sex-specific quartiles: men, <17 years, 17 years, 18 years, and ≥19 years; women, <14 years, 14–15 years, 16–17 years, and ≥18 years. ^§^ Weight when height stopped growing was categorized into sex-specific quartiles: men, <65.8, 65.8–72.5, 72.6–79.4, and >79.4 kg; women, <49.9, 49.9–54.3, 54.4–59.0, and >59.0 kg.

## Data Availability

The full dataset supporting the findings of this study is not publicly available due to privacy and confidentiality concerns. The data contain sensitive, identifiable information and are subject to compliance with the Health Insurance Portability and Accountability Act (HIPAA) in the United States. Data may be made available upon reasonable request to the corresponding author.

## Supplementary Materials

The following supporting information can be downloaded at https://www.mdpi.com/article/10.3390/cancers18162666/s1, Table S1: Frequencies of missingness * by case-control status for self-reported perinatal and early life exposures; Table S2: Characteristics of cases with and without information for breastfeeding duration; Table S3: Characteristics of controls with and without information for breastfeeding duration.
