# Supplementary material for: Perinatal and Early-Life Exposures and Risk of Non-Hodgkin Lymphoma
Source: Cancers (Basel). 2026 Aug 18;18(16):2666. doi: 10.3390/cancers18162666 (PMC13510936; doi:10.3390/cancers18162666)
Supplement: Supplementary file 1 [file cancers-18-02666-s001.zip › Table S1.pdf]

Supplemental Table S1. Frequencies of missingness* by case-control status for self-reported perinatal and early life exposures.

	Control (N=2253)	NHL (N=2280)	Total (N=4533)
**Maternal age**			
Missing	190 (8.4%)	209 (9.2%)	399 (8.8%)
Non-missing	2063 (91.6%)	2071 (90.8%)	4134 (91.2%)
**Birth Order**			
Missing	37 (1.6%)	25 (1.1%)	62 (1.4%)
Non-missing	2216 (98.4%)	2255 (98.9%)	4471 (98.6%)
**Birth Weight**			
Missing	997 (44.3%)	1025 (45.0%)	2022 (44.6%)
Non-missing	1256 (55.7%)	1255 (55.0%)	2511 (55.4%)
**Birth type**			
Missing	18 (0.8%)	13 (0.6%)	31 (0.7%)
Non-missing	2235 (99.2%)	2267 (99.4%)	4502 (99.3%)
**Birth type**			
Missing	2212 (98.2%)	2244 (98.4%)	4456 (98.3%)
Non-missing	41 (1.8%)	36 (1.6%)	77 (1.7%)
**Gestational birth**			
Missing	33 (1.5%)	19 (0.8%)	52 (1.1%)
Non-missing	2220 (98.5%)	2261 (99.2%)	4481 (98.9%)
**Eclampsia/preeclampsia**			
Missing	679 (30.1%)	765 (33.6%)	1444 (31.9%)
Non-missing	1574 (69.9%)	1515 (66.4%)	3089 (68.1%)
**Neonatal jaundice**			
Missing	773 (34.3%)	876 (38.4%)	1649 (36.4%)
Non-missing	1480 (65.7%)	1404 (61.6%)	2884 (63.6%)
**Time Breastfed**			
Missing	1043 (46.3%)	1105 (48.5%)	2148 (47.4%)

	Control (N=2253)	NHL (N=2280)	Total (N=4533)
Non-missing	1210 (53.7%)	1175 (51.5%)	2385 (52.6%)
**Relative Height at 7 years**			
Missing	86 (3.8%)	89 (3.9%)	175 (3.9%)
Non-missing	2167 (96.2%)	2191 (96.1%)	4358 (96.1%)
**Relative Height at 12 years**			
Missing	45 (2.0%)	69 (3.0%)	114 (2.5%)
Non-missing	2208 (98.0%)	2211 (97.0%)	4419 (97.5%)
**Relative Height at 18 years**			
Missing	29 (1.3%)	55 (2.4%)	84 (1.9%)
Non-missing	2224 (98.7%)	2225 (97.6%)	4449 (98.1%)
**Relative Weight at 7 years**			
Missing	53 (2.4%)	70 (3.1%)	123 (2.7%)
Non-missing	2200 (97.6%)	2210 (96.9%)	4410 (97.3%)
**Relative Weight at 12 years**			
Missing	39 (1.7%)	53 (2.3%)	92 (2.0%)
Non-missing	2214 (98.3%)	2227 (97.7%)	4441 (98.0%)
**Relative Weight at 18 years**			
Missing	31 (1.4%)	47 (2.1%)	78 (1.7%)
Non-missing	2222 (98.6%)	2233 (97.9%)	4455 (98.3%)
**Age stopped getting taller**			
Missing	535 (23.7%)	578 (25.4%)	1113 (24.6%)
Non-missing	1718 (76.3%)	1702 (74.6%)	3420 (75.4%)
**Weight when stopped getting taller**			
Missing	670 (29.7%)	721 (31.6%)	1391 (30.7%)
Non-missing	1583 (70.3%)	1559 (68.4%)	3142 (69.3%)

*Missingness represents if the patient reported “Don’t know” or if they did not answer the question.
