# Supplementary material for: Perinatal and Early-Life Exposures and Risk of Non-Hodgkin Lymphoma
Source: Cancers (Basel). 2026 Aug 18;18(16):2666. doi: 10.3390/cancers18162666 (PMC13510936; doi:10.3390/cancers18162666)
Supplement: Supplementary file 1 [file cancers-18-02666-s001.zip › Table S2.pdf]

Supplemental Table S2. Characteristics of cases with and without information for breastfeeding duration.

	Available (N=1645)	Missing (N=592)	p-value
Age (years)			< 0.001
Mean (standard deviation)	60.8 (13.5)	61.7 (13.1)	
Median	62.0	63.0	
Q1, Q3	52.0, 71.0	54.0, 71.0	
Range	18.0 - 91.0	18.0 - 91.0	
Sex			< 0.001
Female	746 (45.3%)	930 (41.6%)	
Male	899 (54.7%)	1307 (58.4%)	
Residence			0.285
Iowa	303 (18.4%)	401 (17.9%)	
Minnesota	1107 (67.3%)	1524 (68.1%)	
Wisconsin	219 (13.3%)	294 (13.1%)	
Missing	16 (1.0%)	18 (0.8%)	
Education			0.003
Some high school	366 (22.2%)	536 (24.0%)	
Beyond high school	438 (26.6%)	599 (26.8%)	
College Graduate	598 (36.4%)	772 (34.5%)	
Missing/other	243 (14.8%)	330 (14.8%)	
Mayo Registration (1991)			0.068
≤ 1991	521 (32.7%)	733 (33.8%)	
> 1991	1074 (67.3%)	1437 (66.2%)	
Reported race/ethnicity			0.682
Non-Hispanic White	1618 (98.3%)	2205 (98.6%)	
Non-Hispanic Black	3 (0.2%)	4 (0.2%)	
Non-Hispanic Asian	9 (0.5%)	10 (0.4%)	
Non-Hispanic Other	1 (0.1%)	1 (0.0%)	
Hispanic (all races)	14 (0.9%)	17 (0.8%)	

Family history NHL			0.155
No	1536 (93.6%)	2079 (93.1%)	
Yes	105 (6.4%)	153 (6.9%)	
Missing	4	5	
Smoking history			0.005
Never	906 (56.5%)	1185 (54.5%)	
Former	566 (35.3%)	798 (36.7%)	
Current	132 (8.2%)	193 (8.9%)	
Missing	41	61	
Alcohol history			0.008
Never	408 (25.1%)	519 (23.5%)	
Former	351 (21.6%)	495 (22.4%)	
Current	864 (53.2%)	1197 (54.1%)	
Missing	22	26	
History B-cell autoimmune disease			0.494
No	1504 (92.7%)	2041 (92.4%)	
Yes	119 (7.3%)	167 (7.6%)	
Missing	22	29	
History T-cell autoimmune disease			0.643
No	1554 (95.7%)	2113 (95.6%)	
Yes	70 (4.3%)	98 (4.4%)	
Missing	21	26	
History of any allergy			0.395
No	1195 (72.7%)	1636 (73.2%)	
Yes	449 (27.3%)	600 (26.8%)	
Missing	1	1	
BMI, Young Adult			0.258
≤ 18.5	134 (8.3%)	177 (8.1%)	
18.5-22.5	833 (51.7%)	1108 (50.7%)	
22.5-25	368 (22.9%)	506 (23.2%)	

25-30	237 (14.7%)	337 (15.4%)	
≥ 30	38 (2.4%)	57 (2.6%)	
Missing	35	52	
Sun Exposure (Quart)			0.172
Q1/Q2	686 (48.1%)	907 (47.2%)	
Q3/Q4	740 (51.9%)	1015 (52.8%)	
Missing	219	315	
NHL Subtype			0.115
CLL/SLL	466 (28.3%)	634 (28.3%)	
DLBCL	303 (18.4%)	404 (18.1%)	
FL	388 (23.6%)	520 (23.2%)	
MCL	81 (4.9%)	127 (5.7%)	
MZL	129 (7.8%)	165 (7.4%)	
Other	191 (11.6%)	271 (12.1%)	
TCL	87 (5.3%)	116 (5.2%)	
