# Supplementary material for: Perinatal and Early-Life Exposures and Risk of Non-Hodgkin Lymphoma
Source: Cancers (Basel). 2026 Aug 18;18(16):2666. doi: 10.3390/cancers18162666 (PMC13510936; doi:10.3390/cancers18162666)
Supplement: Supplementary file 1 [file cancers-18-02666-s001.zip › Table S3.pdf]

Supplemental Table S3. Characteristics of controls with and without information for breastfeeding duration.

	Available (N=1665)	Missing (N=565)	p-value
Age (years)			< 0.001
Mean (standard deviation)	60.9 (13.4)	63.9 (12.1)	
Median	63.0	65.0	
Q1, Q3	53.0, 70.0	56.0, 73.0	
Range	18.0 - 93.0	24.0 - 87.0	
Sex			< 0.001
Female	851 (51.1%)	201 (35.6%)	
Male	814 (48.9%)	364 (64.4%)	
Residence			0.344
Iowa	193 (11.6%)	71 (12.6%)	
Minnesota	1317 (79.1%)	453 (80.2%)	
Wisconsin	154 (9.2%)	40 (7.1%)	
Missing	1 (0.1%)	1 (0.2%)	
Education			0.066
Some high school	364 (21.9%)	149 (26.4%)	
Beyond high school	443 (26.6%)	159 (28.1%)	
College Graduate	777 (46.7%)	232 (41.1%)	
Missing/other	81 (4.9%)	25 (4.4%)	
Mayo Registration (1991)			0.008
≤ 1991	981 (59.1%)	368 (65.4%)	
> 1991	680 (40.9%)	195 (34.6%)	
Reported race/ethnicity			0.966
Non-Hispanic White	1629 (97.8%)	555 (98.2%)	
Non-Hispanic Black	5 (0.3%)	2 (0.4%)	
Non-Hispanic Asian	6 (0.4%)	1 (0.2%)	
Non-Hispanic Other	8 (0.5%)	2 (0.4%)	
Hispanic (all races)	17 (1.0%)	5 (0.9%)	

Family history NHL			0.752
No	1600 (96.2%)	540 (95.9%)	
Yes	63 (3.8%)	23 (4.1%)	
Missing	2	2	
Smoking history			0.021
Never	954 (58.7%)	303 (55.5%)	
Former	563 (34.6%)	219 (40.1%)	
Current	109 (6.7%)	24 (4.4%)	
Missing	39	19	
Alcohol history			0.838
Never	324 (19.8%)	117 (20.9%)	
Former	338 (20.6%)	113 (20.2%)	
Current	977 (59.6%)	329 (58.9%)	
Missing	26	6	
History B-cell autoimmune disease			0.379
No	1565 (94.9%)	527 (93.9%)	
Yes	84 (5.1%)	34 (6.1%)	
Missing	16	4	
History T-cell autoimmune disease			0.530
No	1576 (95.5%)	540 (96.1%)	
Yes	75 (4.5%)	22 (3.9%)	
Missing	14	3	
History of any allergy			0.002
No	1091 (65.5%)	411 (72.7%)	
Yes	574 (34.5%)	154 (27.3%)	
BMI at age 18 years			0.590
≤ 18.5	179 (10.9%)	51 (9.2%)	
18.5-22.5	861 (52.5%)	287 (52.0%)	
22.5-25	382 (23.3%)	133 (24.1%)	
25-30	172 (10.5%)	68 (12.3%)	

≥ 30	46 (2.8%)	13 (2.4%)	
Missing	25	13	
Sun Exposure (Quart)			0.897
Q1/Q2	793 (48.7%)	269 (49.0%)	
Q3/Q4	836 (51.3%)	280 (51.0%)	
Missing	36	16	
